# A bird species occurrence dataset from passive audio recordings across dense urban areas in Gothenburg, Sweden

**DOI:** 10.1038/s41597-025-05481-z

**Published:** 2025-07-10

**Authors:** Ahmed Hazem Eldesoky, Jorge Gil, Oskar Kindvall, Ioanna Stavroulaki, Leif Jonasson, David Bennett, Wenqing Yang, Alonso Francisco Martínez Diaz, Rachel Lichter, Frixos Petrou, Meta Berghauser Pont

**Affiliations:** 1 Spatial Morphology Group (SMoG), Department of Architecture and Civil Engineering, Chalmers University of Technology, Gothenburg, Sweden; 2 Calluna AB, Linköping, Sweden; 3 Independent Ornithologist, Gothenburg, Sweden; 4 Department of Landscape Ecology, Institute for Natural Resource Conservation, Christian-Albrechts-Universität zu Kiel, Kiel, Germany; 5 Stadsmiljöförvaltningen, Göteborgs Stad, Gothenburg, Sweden; 6 University of Cyprus, Nicosia, Cyprus

**Keywords:** Biodiversity, Urban ecology, Geography, Environmental social sciences

## Abstract

Bird species occurrence datasets are a valuable resource for understanding the effects of urbanization on various biotic conditions (e.g., species occupancy and richness). Existing datasets offer promising opportunities to explore variations among cities and along the urban-rural gradient. However, there is a lack of observation data to systematically capture intra-urban variations at a fine spatial scale, especially in dense urban areas. Here, we describe the production and validation of a machine learning-generated bird occurrence dataset based on 10,691 hours of passive audio recordings systematically collected across different types of dense urban forms in Gothenburg, Sweden. The dataset is available in a standard Darwin Core Archive (DwC-A) format, to ensure data interoperability, and includes 239,597 occurrence records of 61 species from April 21 to June 16, 2024, across 30 sites in Gothenburg. We anticipate that this dataset will be a valuable resource for researchers in urban ecology, planning, and design to better understand the relationship between the characteristics of different types of dense urban forms and various biotic conditions in cities.

## Background & Summary

To achieve global sustainability goals, planners and decision-makers worldwide have adopted strategies that promote denser and more compact urban areas. While higher densities have positive effects, especially at larger scales, they often have negative effects locally^1^. Among these is the deterioration of biotic conditions, including changes in species occupancy, distribution, diversity, abundance, and interactions, which are crucial for human health and well-being^2^.

Understanding the relationship between higher density and different biotic conditions at the local scale requires collecting species data from within dense urban areas at high spatial and temporal resolution. Of particular importance is species occurrence data, which provide information on the presence or absence of a species (or other taxon) at specific locations and times. The availability of occurrence datasets has greatly increased in recent years, mainly due to the rise of citizen-science platforms that facilitate sharing observation data^3,4^. Among the most widely studied and recorded species in these occurrence datasets are birds^5^. This is because birds are widely distributed, highly diverse, and relatively easy to detect and identify^6^. Furthermore, birds provide key ecosystem services (e.g., pollination, seed dispersal, pest control, and cultural ecosystem services) and are sensitive to changes in the ecosystem, making them excellent study subjects in many areas of ecology^7^.

Nevertheless, existing citizen-science bird (or other species) occurrence datasets have several limitations. Most notably, the datasets are often unstructured, i.e., the data have not been systematically collected or the data collection method is unknown or not well-documented, making them subject to geographical, temporal, and taxonomic biases^3,4^. The latter results in more easily identifiable species being more frequently recorded, exacerbated by a lack of rigorous review and validation^8^. Hence, these datasets are not very effective in capturing intra-urban variations in biotic conditions at fine spatial scales^9^.

To address the aforementioned limitations in existing datasets, especially the geographical and temporal biases, we produced a machine learning (ML) generated bird species occurrence dataset based on 10,691 hours of passively recorded audio data in Gothenburg, Sweden. This dataset is unique in three ways. Firstly, the data were systematically collected across study sites, meaning that the data collection process followed a structured and consistent methodology, ensuring comparability across space and time. Secondly, we focused on collecting data from within dense urban areas to capture intra-urban variations in biotic conditions, rather than the conventionally studied urban-rural gradient^10^. Thirdly, the dataset underwent rigorous validation and post-processing, where an expert ornithologist manually reviewed a sample of the automated species detections to ensure the overall reliability and accuracy of the dataset.

More specifically, this dataset was produced leveraging advances in passive acoustic monitoring (PAM) and ML. PAM relies on passive acoustic recorders (e.g., microphones, hydrophones) to monitor wildlife based on their sound characteristics (e.g., frequency and amplitude)^11^. This approach has been used in several previous studies ^e.g.12–15^ and is promising for monitoring species occurrence for several reasons. Most importantly, it allows for: systematic and standardized collection of data over extended periods (weeks to months); broader geographic coverage; reduced subjectivity and observer bias; less disturbance to species; and monitoring nocturnal or hard-to-detect animals, such as bats^16,17^. PAM has become more popular due to the advances in acoustic recorders that have become affordable, off-the-shelf, autonomous, programmable, and full-spectrum^18^. On the other hand, advances in ML have made detecting animal vocalizations in passively recorded audio data more automated and cost-effective, particularly through the use of image segmentation techniques on labeled spectrograms^19^. However, there are several limitations to this approach. PAM is suitable only for vocalizing animals and primarily provides information on species presence or absence, activity patterns, and diversity. However, some studies have also used PAM to infer population density and abundance based on, for example, vocal activity rates^20,21^. Also, some animal vocalizations can be challenging to distinguish, especially for species with similar calls or those that mimic other species. Furthermore, when used in urban areas, anthropogenic background noise (e.g., from traffic) can interfere with recordings and mask biophony^22^, making it difficult to detect or accurately classify vocalizations^11^. PAM also requires special data storage infrastructure and continuous improvements in the ML classification models in terms of prediction accuracy.

Based on the above background, we anticipate that this dataset will be a valuable resource for researchers in urban ecology, planning, and design, among other urban studies fields. For example, such a dataset can help in better understanding and modeling the relationship between the characteristics of different types of dense urban forms (e.g., in terms of open/green spaces, buildings, streets) and various biotic conditions, such as species occupancy, distribution, activity, richness, interactions, and functional diversity. Furthermore, the temporal coverage of this dataset makes it suitable for phenological studies in an urban context.

In the following section, we provide further methodological details on how the data were collected, processed, validated, and produced in the final format. Next, we provide a detailed description of the data records. Finally, we provide some usage notes on the dataset.

## Methods

**Fig. 1:**
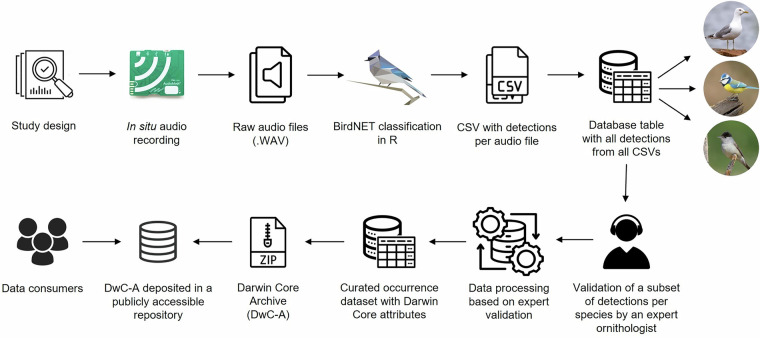
The methodology for producing the bird species occurrence dataset.

The methodology for producing the dataset consisted of four main steps with several sub-steps (Fig. 1): (1) study design; (2) *in situ* deployment, maintenance, and data retrieval; (3) data management, analysis, and wrangling; and (4) technical validation, post-processing, and production of the final dataset and package. These sub-steps are detailed in the following sub-sections.

### Study design

#### Survey timing and site selection (temporal and spatial sampling)

**Fig. 2:**
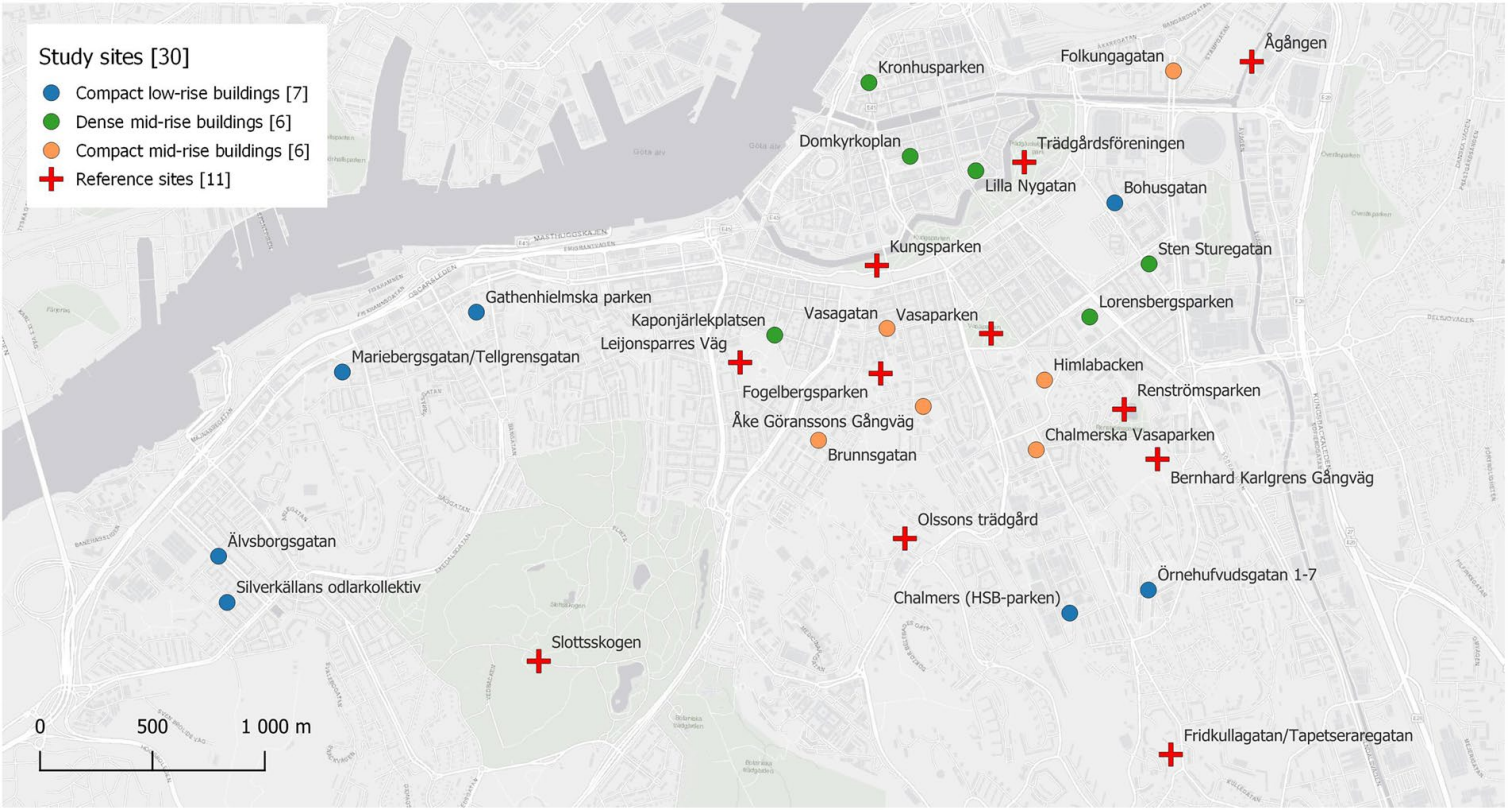
The 30 study sites across central Gothenburg, Sweden.

We collected bird species occurrence data during the 2024 breeding season, when bird vocal activity is at its peak^23^. Specifically, the data were collected from the 21^st^ of April to the 16^th^ of June 2024 across 30 sites in Gothenburg, Sweden (Fig. 2). Of these, 19 sites represented three distinct types of dense urban forms: compact low-rise buildings, compact mid-rise buildings, and dense mid-rise buildings (see Berghauser Pont *et al*.^24,25^ for a detailed description of these types). Acoustic recorders were placed at 6–7 representative sites of each of the three dense urban form types, attached to trees in publicly accessible urban green spaces between buildings or to trees along streets. The other 11 sites were designated as reference sites, i.e., where bird vocalization activity is expected to be higher, such as big urban parks, woodlands, and more spacious urban form types with abundance of vegetation.

#### Approvals and permits

Considering that acoustic recording may raise privacy concerns, before the *in situ* deployment, we acquired ethical approval from Chalmers University of Technology’s Institutional Review Board on Research Ethics (Dr.nr: M2 2023-0194:6D) and conducted a Data Protection Impact Assessment (DPIA). The DPIA outlines the actions taken to minimize data protection risks. This included, for instance: automatically identifying and excluding audio files with human vocalizations; setting a recording schedule with intermittent short clips to minimize the capture of complete conversations (if any occur near the recorders); and making good-faith efforts to inform the public that recording is occurring. Furthermore, we obtained placement permits from the municipality of Gothenburg to legally deploy acoustic recorders on public land.

#### Hardware (acoustic recorder equipment)

The hardware used for the PAM across the 30 sites was the AudioMoth (v1.2.0)^26^, a low-cost (<€100), full-spectrum (up to 192 kHz) acoustic recorder with a small size (5.8 × 4.8 × 1.5 cm). It records uncompressed WAV files to a microSD card to preserve audio quality and is powered by three AA batteries. The AudioMoths were housed in waterproof injection-molded cases (IPX7) that were tested to ensure sound quality. A detailed guide on getting the necessary supplies (e.g., microSD cards, batteries, cases) and assembling and configuring an AudioMoth can be found, for example, in Rhinehart and Guzman^27^.

#### Recording configuration

Each AudioMoth was configured to record at a sampling rate of 96 kHz with a medium gain setting (i.e., a boost in amplitude applied to the audio signal). A sampling rate of at least twice the highest call frequency of interest, known as the Nyquist frequency, is required to resolve all frequency information^11^. Determining the optimal gain typically requires experimenting with different gain settings, depending on the field conditions^28^. However, in most cases, a medium gain setting is appropriate to capture quieter sounds at good quality while minimizing the risk of clipping or excessive background noise, especially in urban areas^29^. For privacy protection, battery life, data storage, and computational considerations, we employed a targeted “on-off” time sampling approach focused on the times of peak bird vocal activity (around the main dawn, morning, and evening chorus times). Specifically, the AudioMoths were configured to record for 1 minute every 2 minutes over a total of 12 hours per day (3 hours before sunrise to 4 hours after, and 2 hours before sunset to 3 hours after). This approach was found to capture data highly comparable to continuous recordings while avoiding an overwhelming data mountain^30,31^.

#### Pilot sampling

Prior to the large-scale deployment, we conducted smaller-scale pilots at a subset of the planned sites to test and confirm the aforementioned recording configurations, deployment conditions, and analysis methods.

### *In situ* deployment, maintenance, and data retrieval

**Fig. 3:**
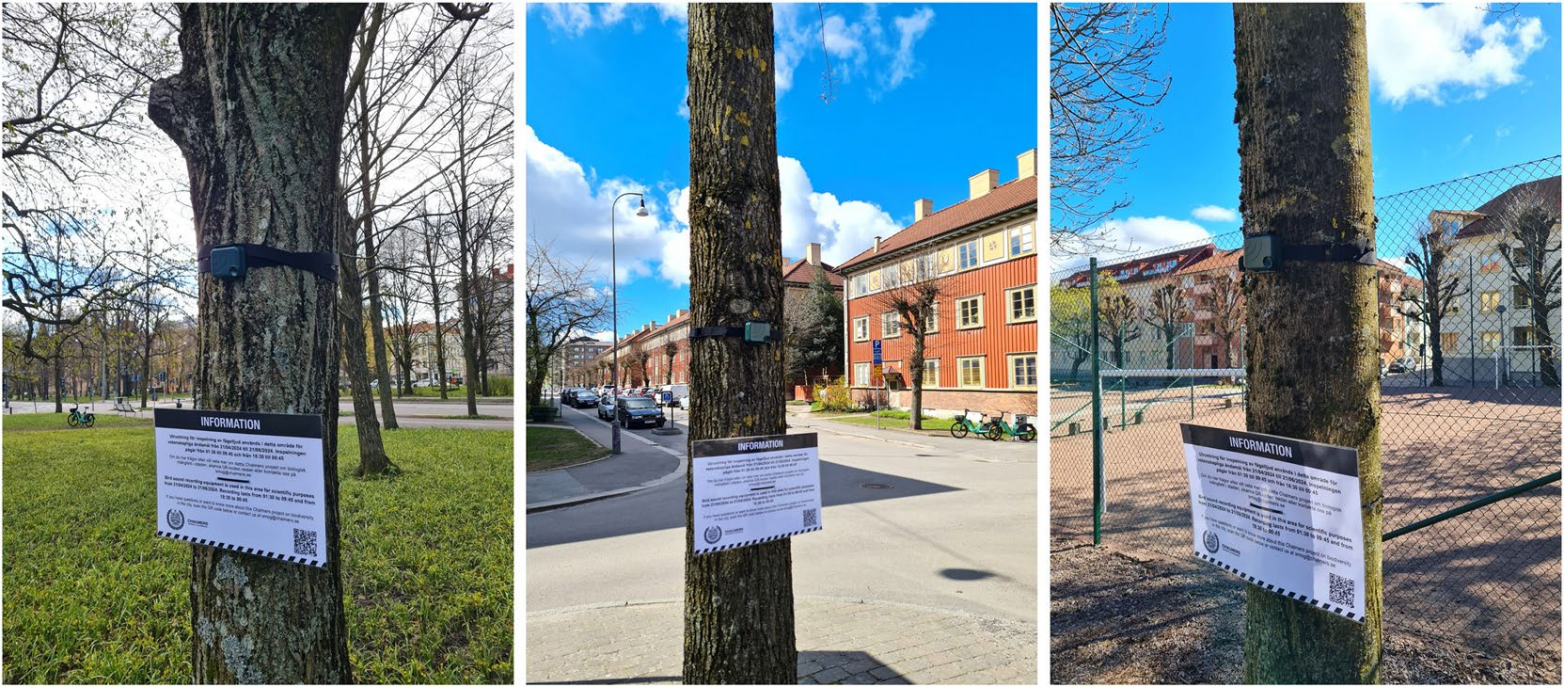
AudioMoth recorders housed in IPX7 waterproof cases and strapped to tree trunks in different study sites in Gothenburg, Sweden.

For each of the 30 sites, recorders were positioned horizontally at around 3 meters above the ground on tree trunks to improve sound quality and protect them from theft or vandalism (Fig. 3). Furthermore, recorders were placed away from branches and leaves as much as possible and oriented to face away from streets to reduce background noise^11,16,27^. In addition to the recorders, signs were placed at each site, in compliance with the DPIA, to inform the public that recording was taking place with more details about the recording times, the purpose of the study, and contact information for inquiries.

Throughout the deployment period, batteries were replaced regularly, and the audio files were retrieved from the microSD cards before they became full. Furthermore, site metadata (e.g., name, latitude, longitude, recorder unique ID, microSD card number, deployment and pickup dates and times, and notes) were recorded during the initial deployment and updated with each subsequent site visit.

### Data management, analysis, and wrangling

In total, we collected 641,502 audio files, amounting to approximately 10,691 hours of recordings and around 7 TB of data. Each audio file was named with date and time information and stored in a directory structure organized by the round of data collection (a total of five rounds), site, and date.

The raw audio files were used as input for a convolutional neural network (CNN) model to identify vocalizing bird species based on 3-second clips/spectrograms, namely BirdNET^19^. BirdNET is a state-of-the-art CNN model that is pre-trained on 6,522 species (v1.3.1, version used) and has shown good accuracy in several studies^32^. The BirdNET model was run in R (v4.4.1) using the NSNSDAcoustics package (https://github.com/nationalparkservice/NSNSDAcoustics) to process the audio files, applying the default inference settings (detection sensitivity of 1.0, no segment overlap, and a minimum confidence threshold of 0.1), and using a custom species list.

Sensitivity controls how responsive BirdNET is to faint or background vocalizations, with higher values leading to more detections. Overlap determines whether each 3-second prediction segment should begin immediately after the previous segment or start earlier, allowing for a temporal overlap between segments. Recently, some studies have shown that increasing sensitivity and/or overlap values from default settings can improve BirdNET’s performance (e.g., by reducing false negatives and thereby increasing recall), particularly for short recording schemes and lower temporal aggregations (e.g., minute-, day-, or week-level)^33–36^. However, with longer and more frequent data collection, as in our case (i.e., recording for 1 minute every 2 minutes over 12 hours per day for two months), this may not be necessary, as bird calls potentially missed due to being cut off at segment edges are likely to recur^34^. Also, higher sensitivity and/or overlap settings can increase the false positive rate, which requires more extensive manual validation^36^. This also significantly increases processing time, which can become a major challenge when processing large amounts of audio data without much improvement in recall^19,35^.

The confidence score is “a unitless, numeric expression of BirdNET’s confidence in its prediction [ranging from 0 to 1]”^37^ ^(p778)^. A higher confidence score indicates a higher likelihood of correct classification^35^. The choice of a minimum confidence score threshold to filter the detections directly impacts the number of species detected, with lower confidence scores resulting in a larger number of species detected^32^. Therefore, selecting an appropriate minimum confidence threshold and manual validation of resulting species detections by an expert ornithologist is essential to ensure the accuracy of the results^38^. To identify the best minimum confidence threshold, we ran the BirdNET analysis using the default low confidence level (0.1) to obtain a complete list of detections, which could then be filtered by testing different confidence thresholds as explained in the next section.

The custom species list used was generated using all publicly available data from the Swedish Species Observation System (Artportalen; https://artportalen.se/) on bird species observed in Gothenburg, Sweden, and included 351 species. Artportalen is the main repository for Swedish species observations and is well acknowledged for its successful gathering of data from ornithologists active in Sweden^39,40^. Using a custom species list—either by supplying BirdNET with location and week-of-year information or by manually compiling a list—is recommended to improve performance, as it helps exclude unlikely species beforehand, reducing false positives^34^. The custom list was first compared to the list of species on which the BirdNET model was trained (6,522 bird and non-bird species) to identify any inconsistencies or missing species. Six bird species had scientific name synonyms, and eight local species were completely missing from BirdNET’s training data: *Gulosus aristotelis*, *Alle alle*, *Glareola nordmanni*, *Polysticta stelleri*, *Anas crecca carolinensis*, *Falco eleonorae*, *Pagophila eburnea*, and *Branta ruficollis*. However, these eight species were deemed unlikely to occur in the study sites when evaluated by an expert ecologist (O.K.). Therefore, the final species list used for analysis included 343 species. In addition to the bird species, we included in the custom species list three labels for human-produced sounds that BirdNET can detect, namely “Human vocal”, “Human non-vocal”, and “Human whistle”. These labels were included to facilitate the identification and exclusion of audio files containing human vocalizations, in compliance with the DPIA mentioned earlier.

The output of the BirdNET model is a CSV file per WAV file, containing a list of detections (rows) and corresponding attributes (columns), such as the path to the processed audio file (named with the date and time information), species’ scientific name, start and end seconds of the detection, and confidence score.

In the next step, the CSV files were merged into a database table with 4,664,290 rows (observations) and a total of 338 unique bird species detected out of the 343 in the custom species list. The dataset was then processed to extract the site, date, and time information from the file path into separate columns and to create a unique recording identifier column for easier data manipulation.

### Technical validation, post-processing, and production of the final dataset

To identify an appropriate minimum confidence threshold for expert validation, we first set a high threshold of 0.85, which resulted in 206 unique bird species (out of the 338 detected at the minimum threshold of 0.1). We then lowered the threshold to 0.80, where 15 additional species were included. These 15 species had only one or two detections, all of which were found to be false positives after an expert ornithologist (L.J.) listened to the recordings, suggesting that a threshold of 0.85 is suitable for this study. However, this does not necessarily mean that the detections of the remaining 132 species with confidence scores below 0.85 are entirely false positives. Therefore, these 132 species were further scrutinized by the expert ecologist on a case-by-case basis to determine which species were likely to occur at the specific study sites. Nine species were identified as such: *Emberiza schoeniclus*, *Carpodacus erythrinus*, *Podiceps cristatus*, *Oenanthe oenanthe*, *Jynx torquilla*, *Linaria cannabina*, *Luscinia luscinia*, *Acrocephalus schoenobaenus*, and *Alauda arvensis*. For each of these nine species, the expert ornithologist listened to a random sample of audio files in which the species was detected (up to 50 per species where available, totaling 442 one-minute recordings). All examined detections were confirmed to be false positives. The dataset used for expert validation, therefore, includes 206 unique species with 250,472 occurrence records (at a minimum confidence of 0.85).

Following Sethi *et al*.^41^, Bick *et al*.^42^, and Fairbairn *et al*.^34^, for each of the 206 species we randomly retrieved 50 unique audio files in which that species was detected with a minimum confidence of 0.85, ensuring that no two detections of the same species selected for validation came from the same audio file. If a species was detected fewer than 50 times or there were not 50 unique audio files, all available audio files were retrieved. This resulted in a validation dataset of 5,007 one-minute audio files. The expert ornithologist then listened to each audio file and classified each associated detection as correct, incorrect, or unsure. For records classified as incorrect or unsure, the ornithologist, when confident, provided suggestions for the likely species or sound. The ornithologist-validated dataset was used to reclassify and/or remove records and to train a predictive classification model to produce the final curated dataset, as explained in more detail below.

**Fig. 4:**
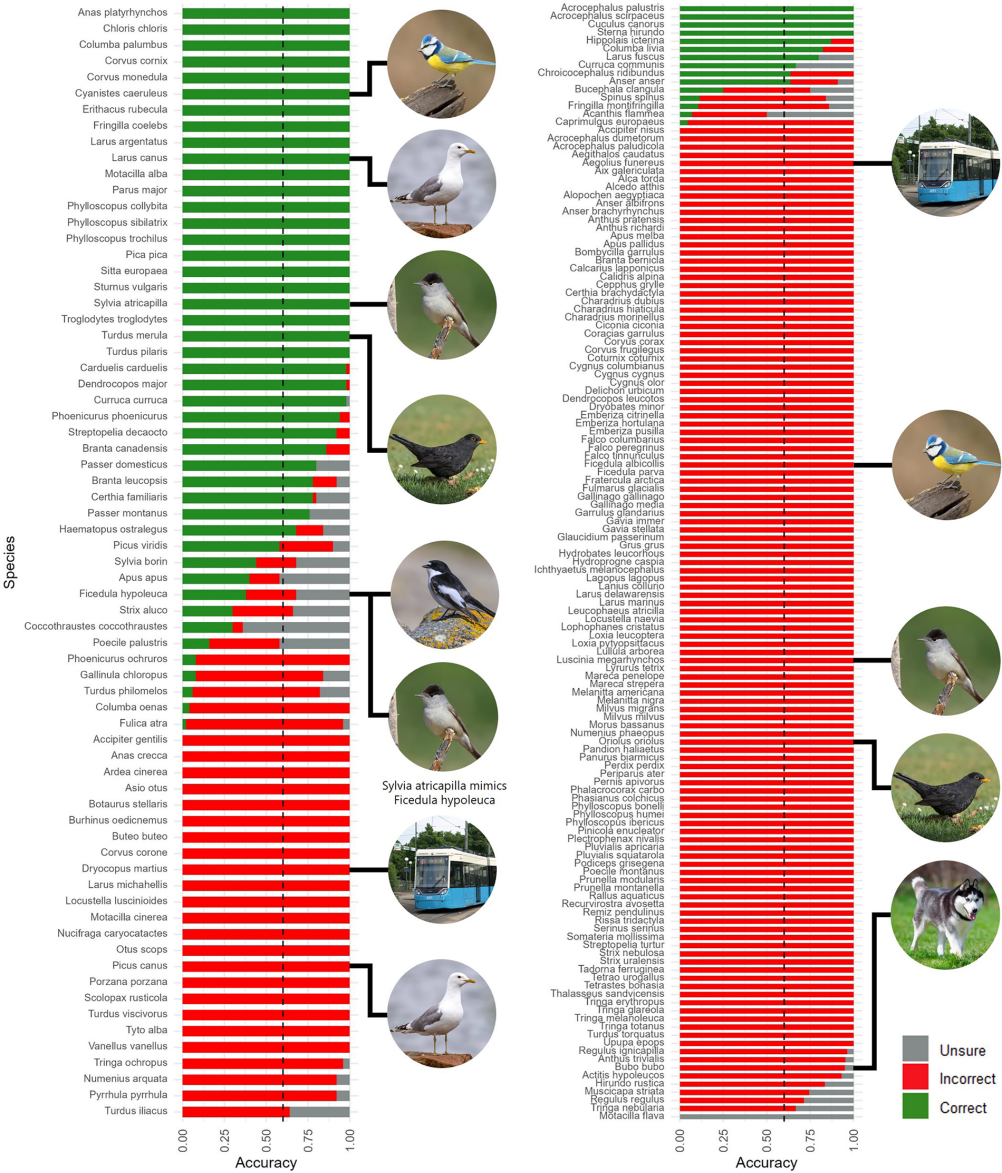
Ornithologists’ validation of BirdNET’s classification results. The figure shows the percentage of correctly classified detections per species (with 50 validated detections, left, and <50 validated detections, right). The vertical dashed lines mark a 60% accuracy threshold. The figure style is adapted from Sethi *et al*.^41^.

The validation results (Fig. 4) show that 43 species achieved a classification accuracy of over 60% (with 26 species having a 100% accuracy), 17 species had an accuracy between 60% and 2%, while all the remaining 146 species were false positives.

**Fig. 5:**
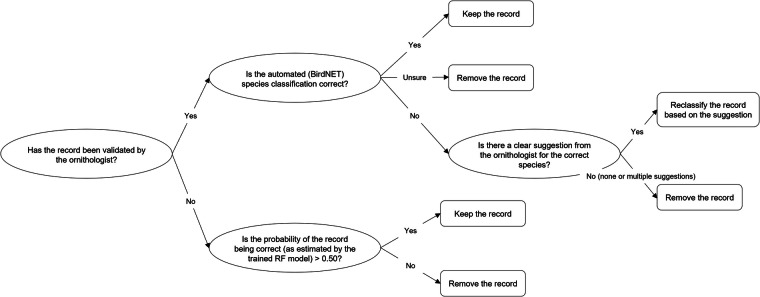
Decision tree for the data records exclusion or reclassification based on the validation results.

Based on the validation results, we further processed the dataset (with 250,472 records) as follows. For the 5,007 “validated” records by the ornithologist, we retained those that were correctly classified, reclassified records that were incorrectly classified and the ornithologist suggested other species, and removed records that were incorrectly classified and the ornithologist could not recognize the species/sound or was unsure (Fig. 5).

For the “non-validated” records, we trained a binary Random Forest (RF) probabilistic classifier based on the ornithologist-validated dataset to estimate the probability that each record was correctly classified by BirdNET. The model training and evaluation process is described in more detail below. In the final dataset, we retained only non-validated records with an estimated probability above 50% (Fig. 5). This process resulted in a curated dataset with 239,597 occurrence records of 61 species. This includes 60 species that achieved an accuracy above zero (Fig. 4), plus one species (i.e., *Regulus regulus*), which the ornithologist identified during validation to be the correct classification for a false positive detection of another species (i.e., *Regulus ignicapilla*).

The RF classifier model was implemented in R using the *ranger* package^43^ (v0.17.0). It was configured with 500 decision trees and trained on 2,496 ornithologist-validated records from the 60 species that achieved an accuracy above zero (Fig. 4). Three predictors were used: (1) species name (BirdNET classification); (2) BirdNET confidence score; and (3) whether the detection was isolated. The latter indicates whether a bird detection is not preceded or followed by another detection of the same species within a 9-second window. Isolated detections have been found to increase false positives^12,44^.

**Fig. 6:**
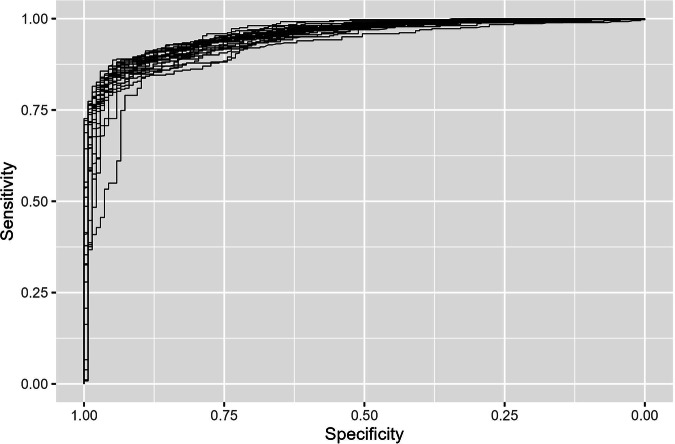
ROC curves from all 25 cross-validation sets (5 folds, repeated 5 times). The average AUC across all curves is 0.95.

To evaluate the model performance and select the best-performing RF model hyperparameters—specifically the number of variables considered at each split (*mtry*) and the node splitting rule (*splitrule*)—we used repeated cross-validation (5 folds, repeated 5 times). Model performance was measured using the area under the ROC (receiver operating characteristic) curve (AUC), a threshold-invariant metric widely used for evaluating binary classifiers^45^. The hyperparameter configuration with the highest average AUC across all cross-validation sets was selected. The ROC curve shows the model’s performance in terms of sensitivity (true positive rate) and specificity (1 − false positive rate) across all possible probability thresholds, with AUC values closer to 1 indicating better model performance in terms of distinguishing between classes. Figure 6 shows the ROC curves from all the cross-validation sets. The best-performing RF model hyperparameter configuration (*mtry* = 2, *splitrule* = gini) resulted in an average AUC of 0.95 across all cross-validation sets. This indicates a high model’s ability to distinguish between the positive and negative classes. The best-performing hyperparameter configuration was used to train the final model on the entire training dataset (2,496 records), and the trained model was then used to estimate probabilities for the non-validated records of 60 species.

In the final step, the curated dataset was further processed by renaming existing attributes and creating new ones in accordance with the Darwin Core standard^46^ (see next section).

## Data Records

The data package associated with this paper is available on Zenodo (version 2.0.0)^47^. The main data product in the package is a bird species occurrence file in a comma-delimited format. The file has 239,597 rows and 30 columns, where each row represents a detection and each column represents an attribute (or “term” as described by the Darwin Core standard). A list and description of the Darwin Core and custom attributes included in the occurrence dataset are provided in Table S1 and are also available online with more information at (https://smog-chalmers.github.io/BirdMonitoringGothenburg/).

In addition to the occurrence dataset, the data package includes the following files:

1. **eml.xml**: A metadata descriptor for the dataset with information about the dataset type, creators, license, and other metadata in Ecological Metadata Language (EML) format^48^. The eml file was produced using the Integrated Publishing Toolkit (IPT), developed by the Global Biodiversity Information Facility (GBIF).
2. **meta.xml**: A file that describes how the files in the DwC archive are organized and specifies the Uniform Resource Identifiers (URIs) for the standard Darwin Core and custom attributes used in the occurrence dataset.
3. **species_accuracy_and_misclassification.txt**: A supplementary table that includes the list of the 206 species validated by the ornithologist. For each species, the table includes its classification accuracy (based on the validated sample of detections) and the probabilities of it being misclassified as other species.
4. **species.txt**: A list of the scientific names of the 61 species included in the occurrence dataset, along with their taxon IDs (i.e., unique Life Science Identifiers or LSIDs) according to the Swedish taxonomic database (Dyntaxa; dyntaxa.se).

## Usage Notes

In this paper, we described the production and validation of an ML-generated bird occurrence dataset in Gothenburg, Sweden, based on 10,691 hours of passively recorded audio data. The dataset offers opportunities for researchers in urban ecology, planning, and design to systematically study intra-urban variations in biotic conditions at a fine spatial scale, especially in dense urban areas. However, the users of this dataset should note the following.

Firstly, the representative spatial area coverage of each acoustic recorder is not an intrinsic characteristic of the recorder itself, it depends on the sound’s amplitude and frequency as well as on other environmental (e.g., temperature, humidity) and site-specific factors (e.g., building density)^11^. Nevertheless, previous studies suggest that the effective detection radius of most recorders is in the range of 50 meters^16,49^. Through playback experiments, Sethi *et al*.^14^ found that maximum detection distances for common sound amplitudes and frequencies ranged from 30–70 meters in open semi-natural settings (grasslands) to 20–30 meters in dense environments (forests).

Secondly, the dataset has a few temporal gaps due to some operational challenges, including an in-house sensor re-configuration period (from May 23 to 26, 2024), depleted batteries in some sites before the next site re-visit (with a maximum gap of one day between each of the five data collection rounds), and equipment loss, where one recorder was stolen from a reference site (Bernhard Karlgrens Gångväg) during the last round of deployment (from June 7 to 16, 2024).

Thirdly, although we have conducted a rigorous technical validation and post-processing of the dataset, reclassifying or excluding occurrence records to enhance its overall accuracy (i.e., the proportion of correct occurrence records in the dataset), we did not conduct a species-specific recall analysis (i.e., the proportion of actual vocalizations by a species that are correctly detected by BirdNET). The latter is particularly important for detailed studies focusing, for example, on species-specific behaviors^32,50^.

**Fig. 7:**
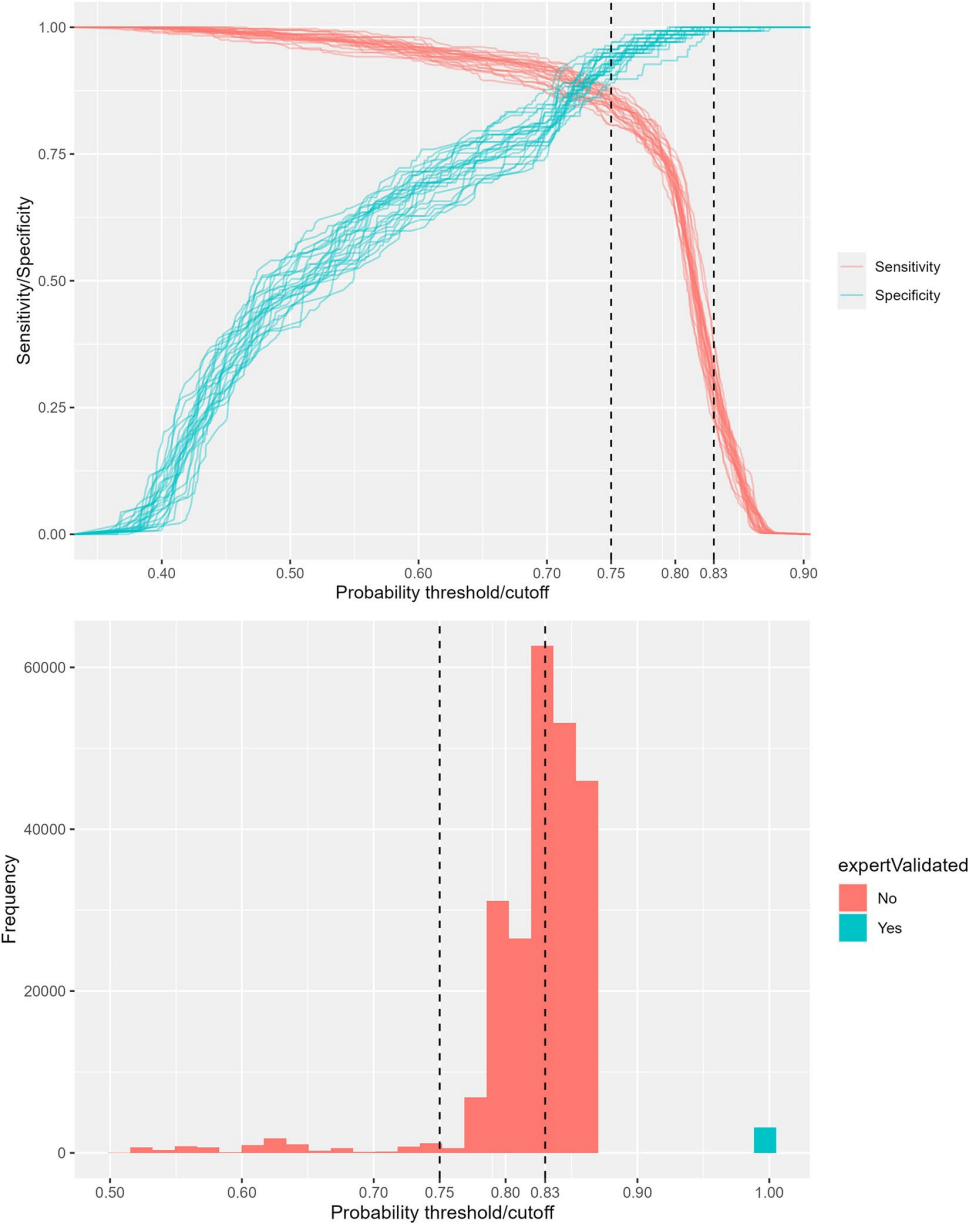
Top: Sensitivity and specificity plotted against probability thresholds for all 25 cross-validation sets (5 folds, repeated 5 times). Bottom: Frequency distribution of occurrence records in the dataset across different probability thresholds. The vertical lines mark the averaged probability thresholds that either balance sensitivity and specificity (0.75) or maximize specificity (0.83).

Fourthly, since the accuracy of the “non-validated” records in the dataset depends on the user-selected threshold applied to the RF probability estimates, we identified threshold values to accommodate different user needs, depending on whether sensitivity or specificity is prioritized. For each of the 25 cross-validation datasets (5 folds, 5 repeats), we extracted: (1) the threshold that balances sensitivity and specificity based on Youden’s J statistic^51^; and (2) the threshold that maximizes specificity. The resulting thresholds were averaged across the 25 cross-validation sets to provide more representative threshold values: 0.75 for balanced sensitivity-specificity and 0.83 for maximum specificity, as shown in Fig. 7 (top). The figure (bottom) also shows the frequency distribution of occurrence records in the dataset across different probability thresholds.

Finally, as outlined in the introduction, species occurrence datasets produced using PAM may exhibit taxonomic bias, as some species present but not vocalizing (or vocalizing infrequently) might be missed. However, this limitation is not unique to PAM and all wildlife monitoring techniques have their own limitations (e.g., hibernating species are challenging to detect by camera traps)^14,52^. Therefore, combining data from different resources and employing multiple monitoring techniques is essential to reduce taxonomic bias in occurrence datasets^53,54^.

## Supplementary information

Description of the attributes used in the dataset.

## Data Availability

The CNN model (BirdNET) used to analyze the collected audio data in this study was developed by the K. Lisa Yang Center for Conservation Bioacoustics at the Cornell Lab of Ornithology at Cornell University, USA. BirdNET models and scripts are available at (https://github.com/kahst/BirdNET-Analyzer) and can be run, for example, through the command-line interface (CLI), a graphical user interface (GUI), application programming interfaces (APIs), and Python scripts for diverse use cases. They can also be run, and the results can be analyzed, in R using the NSNSDAcoustics package available at (https://github.com/nationalparkservice/NSNSDAcoustics). The code produced to analyze the audio files, process the raw BirdNET results to tidy data, retrieve and validate a sample of the detections, and produce the final occurrence dataset in Darwin Core standard can be accessed at (https://github.com/ahmaeldesoky/bird-species-detection-gothenburg-2024).
