# Supplementary material for: A bird species occurrence dataset from passive audio recordings across dense urban areas in Gothenburg, Sweden
Source: Sci Data. 2025 Jul 10;12:1180. doi: 10.1038/s41597-025-05481-z (PMC12246040; doi:10.1038/s41597-025-05481-z)
Supplement: Supplementary file 1 — Description of the attributes used in the dataset. [file 41597_2025_5481_MOESM1_ESM.pdf]

Table S1. Description of the attributes used in the dataset.

**Attribute**	**Description**
**Required Darwin Core attributes**	
occurrenceID	A Universally Unique Identifier (UUID) for the occurrence.
basisOfRecord	The type of the record (i.e., MachineObservation).
scientificName	The full scientific name, with author and date information if known, after reclassifying and excluding records based on the technical validation. The name follows the scientific name currently valid for the taxon according to the Swedish taxonomic database (dyntaxa.se).
eventDate	The date during which the occurrence record was recorded, following the ISO 8601 date-time standard.
**Other Darwin Core attributes (not required)**	
eventTime	The local time interval during which the event occurred, following the ISO 8601 date-time standard.
decimalLatitude	The latitude (in decimal degrees) of the acoustic recorder's location.
decimalLongitude	The longitude (in decimal degrees) of the acoustic recorder's location.
geodeticDatum	The coordinate reference system used for the location (i.e., EPSG:4326).
country	The name of the country of the occurrence record (i.e., Sweden).
countryCode	A two-letter standard abbreviation for the country of the occurrence record (i.e., SE).
taxonRank	The taxonomic rank of the most specific name in the scientificName (i.e., species).
kingdom	The full scientific name of the kingdom in which the taxon is classified (i.e., Animalia).
phylum	The full scientific name of the phylum or division in which the taxon is classified.
class	The full scientific name of the class in which the taxon is classified.
order	The full scientific name of the order in which the taxon is classified.
family	The full scientific name of the family in which the taxon is classified.
genus	The full scientific name of the genus in which the taxon is classified.

Table S1. *Cont.*

Attribute	Description
**Other Darwin Core attributes (not required)**	
taxonID	The unique LSID (Life Science Identifier) of the taxon according to the Swedish taxonomic database (dyntaxa.se) provided by the Swedish Species Information Center (Artportalen).
**Custom attributes**	
globalSortOrder	A taxon-specific attribute provided by the Swedish Species Information Centre (Artportalen). It is an integer value, which can be used to enable a taxonomic sort order of all Swedish taxa handled in the Swedish taxonomic database (dyntaxa.se).
BirdNETClass	The taxon scientific name originally classified by the BirdNET model, prior to the technical validation and reclassification of records.
BirdNETConfidence	The BirdNET built-in confidence score for species detection, ranging from 0 to 1[37]. The dataset includes only species detections with a minimum confidence score of 0.85.
expertValidated	Indicates whether the occurrence record has been validated by an expert ornithologist.
isIsolated	Indicates whether a bird detection is isolated, i.e., not preceded or followed by another detection of the same species within a 9-second window (at a model's detection sensitivity of 1.0, no segment overlap, and a minimum confidence threshold of 0.1).
reclassified	Indicates whether the occurrence record has been reclassified from the original BirdNET classification, following the decision tree shown in Figure 5.
occurrenceProbability	An estimation of the likelihood of a species occurrence, based on expert validation and model predictions, where:<br>• **occurrenceProbability = 1.00**: For expert-validated records (expertValidated == "Yes") that were confirmed to be correct or reclassified based on a clear suggestion from the ornithologist (reclassified == "Yes").<br>• **0.5 < occurrenceProbability < 1.00**: For non-validated records (expertValidated == "No"), this value shows the model-predicted probability of occurrence, based on the trained RF classifier.

Table S1. *Cont.*

Attribute	Description
**Other Darwin Core attributes (not required)**	
commonNameSwedish	The common Swedish name of the taxon.
commonNameEnglish	The recommended common English name of the taxon according to the Swedish taxonomic database (dyntaxa.se).
detectionDistanceInMeters	The expected radius, in meters, around the acoustic recorder within which sounds were detected. The values (i.e., 20–70) are suggested based on the maximum detection distances for typical sound amplitudes and frequencies observed in playback experiments by Sethi et al.[14].
siteName	The name of the site where the recorder was located.
bcl	The building type surrounding the recorder, as described in Berghauser Pont et al.[24,25], where:<br>• bcl2: Compact low-rise buildings<br>• bcl3: Dense mid-rise buildings<br>• bcl5: Compact mid-rise buildings<br>• ref_bcl6: Reference spacious mid-rise buildings<br>• ref: Reference non-built green space
